# Tau Protein Hyperphosphorylation and Aggregation in Alzheimer’s Disease and Other Tauopathies, and Possible Neuroprotective Strategies

**DOI:** 10.3390/biom6010006

**Published:** 2016-01-06

**Authors:** Goran Šimić, Mirjana Babić Leko, Selina Wray, Charles Harrington, Ivana Delalle, Nataša Jovanov-Milošević, Danira Bažadona, Luc Buée, Rohan de Silva, Giuseppe Di Giovanni, Claude Wischik, Patrick R. Hof

**Affiliations:** 1 Department of Neuroscience, Croatian Institute for Brain Research, University of Zagreb School of Medicine, Zagreb 10000, Croatia; mbabic@hiim.hr (M.B.L.); njovanov@hiim.hr (N.J.-M.); 2 Reta Lila Weston Institute and Department of Molecular Neuroscience, UCL Institute of Neurology, London WC1N 3BG, UK; selina.wray@ucl.ac.uk (S.W.); r.desilva@ucl.ac.uk (R.S.); 3 School of Medicine and Dentistry, University of Aberdeen, Aberdeen AB25 2ZD, UK; c.harrington@abdn.ac.uk (C.H.); cmw@taurx.com (C.W.); 4 Department of Pathology and Laboratory Medicine, Boston University School of Medicine, Boston 02118, MA, USA; idelalle@bu.edu; 5 Department of Neurology, University Hospital Center Zagreb, Zagreb 10000, Croatia; b.danira@gmail.com; 6 Laboratory Alzheimer & Tauopathies, Université Lille and INSERM U1172, Jean-Pierre Aubert Research Centre, Lille 59045, France; luc.buee@inserm.fr; 7 Department of Physiology and Biochemistry, Faculty of Medicine and Surgery, University of Malta, Msida, MSD 2080, Malta; giuseppe.digiovanni@um.edu.mt; 8 School of Biosciences, Cardiff University, Cardiff CF10 3AX, UK; 9 Fishberg Department of Neuroscience, Ronald M. Loeb Center for Alzheimer’s Disease, Icahn School of Medicine at Mount Sinai, New York, NY 10029, USA; patrick.hof@mssm.edu; 10 Friedman Brain Institute, Icahn School of Medicine at Mount Sinai, New York, NY 10029, USA

**Keywords:** Alzheimer’s disease, amyloid β, neurofibrillary degeneration, microtubules, neuropathology, phosphorylation, protein aggregation, protein oligomerization, tauopathies, tau protein

## Abstract

Abnormal deposition of misprocessed and aggregated proteins is a common final pathway of most neurodegenerative diseases, including Alzheimer’s disease (AD). AD is characterized by the extraneuronal deposition of the amyloid β (Aβ) protein in the form of plaques and the intraneuronal aggregation of the microtubule-associated protein tau in the form of filaments. Based on the biochemically diverse range of pathological tau proteins, a number of approaches have been proposed to develop new potential therapeutics. Here we discuss some of the most promising ones: inhibition of tau phosphorylation, proteolysis and aggregation, promotion of intra- and extracellular tau clearance, and stabilization of microtubules. We also emphasize the need to achieve a full understanding of the biological roles and post-translational modifications of normal tau, as well as the molecular events responsible for selective neuronal vulnerability to tau pathology and its propagation. It is concluded that answering key questions on the relationship between Aβ and tau pathology should lead to a better understanding of the nature of secondary tauopathies, especially AD, and open new therapeutic targets and strategies.

## 1. Selective Overview of Major Discoveries on Tau Protein and Tauopathies

### 1.1. Neurofibrillary Tangles and Paired Helical Filaments

The Bavarian psychiatrist Aloysius (Alois) Alzheimer is credited with the first description of the most characteristic pathological brain change—neurofibrillary tangles (NFT)—of a yet-unnamed disease in a 51-year-old woman from Frankfurt am Main, who had developed dementia. That woman was the first person to receive a diagnosis of the disease for which in 1910 Emil Kraepelin coined the term Alzheimer’s disease (AD; which he wrongly, albeit cautiously, initially described as “presenile dementia”). Her name was Auguste Deter and she had an early-onset dementia, comorbid with psychotic features. As she became progressively worse, she had to be admitted to a psychiatric hospital in November 1901 (where Alzheimer examined her for the first time), where she eventually died in April 1906. Besides the already known “miliary foci” of extracellular deposits scattered over the cerebral cortex (more commonly later called senile plaques, SP, or neuritic plaques, NP), by using a newly developed silver staining method (20% water solution of silver nitrate, [1]) Alzheimer observed degenerating cortical neurons with bundles of intracellular fibrils (neurofibrillary tangles, NFT) [2,3].

It was not until 1963 that with the help of electron microscopy, Kidd and Terry independently reported NFT to be made up of abnormal filaments alternating between 15 (at their narrowest point) and 30 nm (at their widest point) in width, with a half-periodicity of about 80 nm [4,5]. Because it appeared that the two filaments were wound helically around one another, Kidd named them paired helical filaments (PHF). Also found in NFT of AD, as a minority species, was the so-called straight filament (SF), a filament about 15 nm wide that does not exhibit the marked modulation in width shown by the PHF. Due to the fact that PHF were observed to be insoluble in denaturing agents such as sodium dodecyl sulfate (SDS) and urea, despite significant efforts the structural and molecular composition of PHF (and NFT) was not elucidated until the mid-1980s [6,7]. Morphological studies of fragmentation patterns showed that the PHF actually consists of a left-handed helical ribbon consisting of repeating symmetrical subunits. Using electron diffraction, Crowther and Wischik were able to establish conclusively that the PHF is made up of a double helical stack of transversely oriented C-shaped subunits, each of which has three domains. This structure precluded purely descriptive models available to that point based on rearrangements of preformed cytoskeletal polymers or protofilaments. They concluded that the structure was of a type that might arise from the *de novo* assembly of a single structural subunit, the biochemical identity of which was then unknown. Later studies showed that SF were composed of a similar structural subunit although with a slightly different relative arrangement in the two types of filaments [8].

### 1.2. Tau Protein Isolation and Localization

Tau (tubulin-associated unit) protein was isolated from porcine brain extracts as a heat-stable, highly soluble protein essential for microtubule (MT) assembly [9]. Following the initial discovery of tau, two studies reported the process of tau purification and its physical and chemical properties [10,11], including the ability of tau to become phosphorylated. In 1983, it was discovered that tau could be phosphorylated at multiple sites by various protein kinases, including cyclic-AMP-dependent protein kinases and casein kinase type-1 [12]. Further studies showed that tau is a phosphoprotein and that phosphorylation negatively regulates its ability to stimulate MT assembly [13,14].

An immunohistochemical study that compared the localization of tau using the tau-1 antibody (that recognizes all isoforms of tau, see below) with that of microtubule-associated protein 2 (MAP2) and tubulin in human postmortem brain tissue demonstrated that tau protein was primarily localized to axons [15]. Using the same tau-1 monoclonal antibody and electron microscopy with colloidal gold-labeled secondary antibodies, tau was also found in very low amounts in astrocytes [16] and oligodendrocytes [17], and this was confirmed by tau mRNA expression analysis in the mouse brain [18].

### 1.3. Tau in Neurofibrillary Tangles

The insolubility of PHF precluded biochemical characterisation of the repeating subunit that makes up the structural core of the filament. What was required was a means of solubilising or releasing the structural subunit as a protein band that could be visualised by gel electrophoresis and linking this by immuno-electron microscopy to the PHF. Initial attempts based on relatively crude preparations of NFT were unable to distinguish between proteins copurifying with NFTs due to trapping and loose association within the dense filament bundles, and proteins derived from the structural core of the PHF. In 1985 Brion and collaborators prepared tau and MAP2 proteins from the adult rat brain using the microtubule assembly-disassembly method and their property of thermostability; they then generated antisera against tau and MAP2 proteins using polypeptides extracted from polyacrylamide gels after electrophoretic separation by sodium dodecyl sulfate-polyacrylamide gel electrophoresis (SDS-PAGE) [19]. Antisera were characterized by immunoblotting on purified preparations of tau and MAP2 and found to react with their cognate antigens. These antisera were then used for immunocytochemistry on tissue sections from control subjects and AD patients: the anti-MAP2 antibody did not label NFT but the anti-tau antibody strongly immunolabelled NFT and abnormal neurites around senile plaques, yielding an immunolabelling indistinguishable from the one obtained with anti-PHF serum [19]. This work therefore established that tau protein was one of a number proteins associated with NFTs both histologically and in crude NFT extracts. Neurofibrillary tangles can be labeled histologically with antibodies against a variety of other neuronal proteins, including vimentin, actin, ubiquitin, MAP2, and Aβ protein. In crude NFT preparations, isolated NFT could be labeled with antibodies against MAP2, neurofilament, ubiquitin and tau [19,20,21,22,23,24,25,26,27,28,29].

The proof that tau protein contributes to the structural core of the PHF required preparation of fractions highly in enriched in proteolytically stable PHF which retained the subunit structure of the filament that had been characterised previously. These PHF were solubilised in formic acid and when examined by SDS-PAGE gel electrophoresis were found to contain predominantly a 12-kD protein and a corresponding dimer. Surprisingly, this protein was not recognised by an antibody raised against tau protein. Conversely, a monoclonal antibody raised against the enriched core PHF preparations (mAb 6.423, referred to as MN423) did not recognised purified tau protein [30,31,32,33]. Nevertheless, MN423 was shown by immunogold electron microscopy to label the proteolytically stable core of the PHF. Furthermore, a ligand related to primulin [34] was used to affinity label the 12-kD species. This ligand, when bound covalently to biotin, was also shown by immunogold electron microscopy to label proteolytically stable core PHF. Therefore, the provenance of the 12-kD protein from the structural core of the PHF was unequivocally established by two independent approaches. Partial amino acid sequences derived from this band were unrelated to any protein sequence known at that time. However, when these were used to clone and sequence the corresponding cDNAs from a human brain library, the predicted protein was found to be 352 amino acids in length and was found to have extensive homology to the sequence of the mouse microtubule-associated protein tau isoform that had just been published [35,36]. It was concluded that this protein must constitute the human homolog of mouse tau, and that tau protein therefore must contribute to the structural core of the PHF, and was not simply a loosely associated protein copurifying with NFT. These data were therefore able to explain the earlier observations linking tau protein with NFT [21,37,38,39,40,41]. In the same year tau cDNA clones were identified in the human fetal brain by flow sorting and spot-blot hybridization and later on assigned to the microtubule-associated protein tau gene (*MAPT*) on the long arm of the chromosome 17 [42,43].

### 1.4. Tau Isoforms in the Central Nervous System

**Figure 1:**
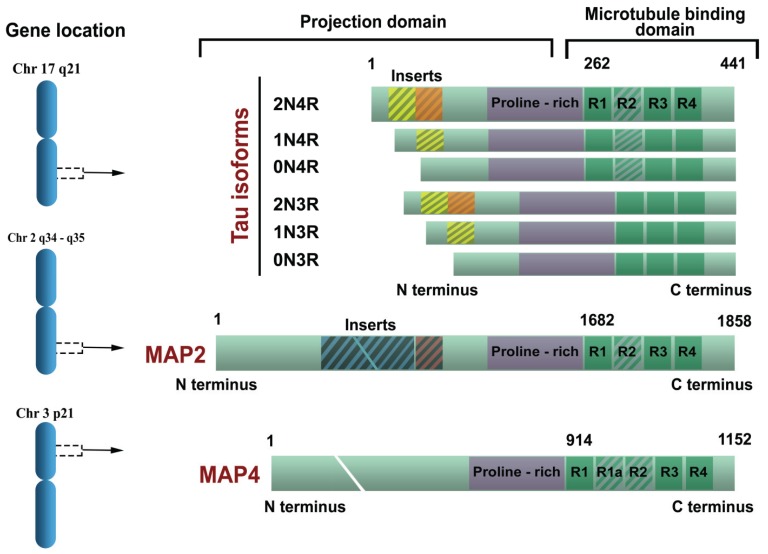
Chromosomal location of the gene and protein structure for the microtubule-associated proteins tau, microtubule-associated protein 2 (MAP2) and MAP4. Tau exons 2, 3 and 10 are alternatively spliced, giving rise to six different mRNAs, translated in six different tau isoforms. Tau isoforms differ by the absence or presence of one or two 29 amino acid inserts encoded by exon 2 (yellow) and 3 (orange) in the N-terminal part, in combination with either three (R1, R3 and R4) or four (R1-R4) repeat regions in the C-terminal part. The R2 repeat is encoded by exon 10. The longest 2N4R adult tau isoform (2+3+10+) has 441 amino acids (aa), followed by 1N4R isoform of 412 aa (2+3−10+), 2N3R isoform of 410 aa (2+3+10−), 0N4R isoform of 383 aa (2−3−10+), 2N3R isoform of 381aa (2+3−10−) and the shortest 0N3R isoform of 352 aa (2−3−10−). The single neuron-specific promoter of *MAPT* gene has three binding sites for transcription factors and its activity increases with axon initiation and outgrowth. The shortest tau isoform is the only one expressed in the fetal brain (“fetal tau”), while expression of other isoforms begins postnatally (for a review, see [48]). The MAP2 and MAP4 have comparable repeat domain sequences in the C-terminus but differ from tau proteins by their longer N-terminal projection arms.

**Figure 2:**
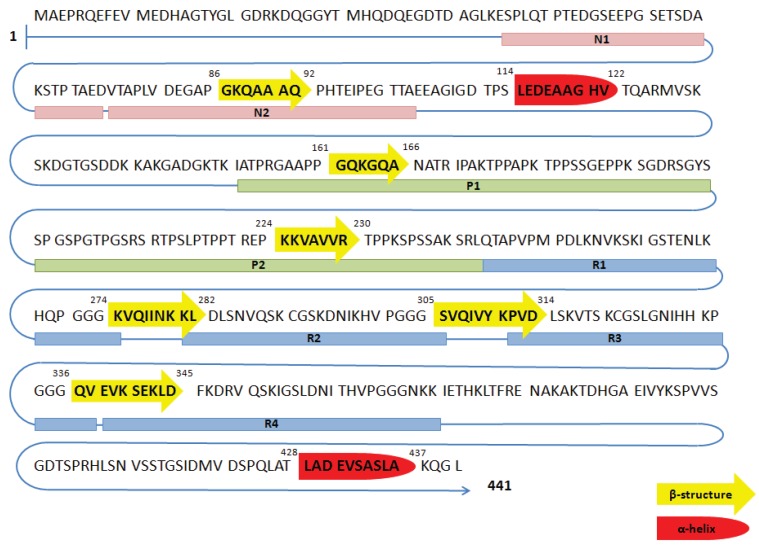
Primary sequence of amino acids and probable secondary structure of the longest tau isoform in the central nervous system. N1 and N2 denote the sequences encoded by exons 2 and 3, respectively. R1 through R4 are microtubule-binding domains encoded by exons 9–12, respectively. Domains with β-sheet structure and α-helical content are shown in yellow and red, respectively.

In their 1988 paper, Goedert and collaborators also mentioned that they had identified a second form of tau, with sequence variation in the first repeat, and suggested that tau mRNA was undergoing alternative splicing [34]. This second form was identical to the first, with the exception of an additional insert of 31 amino acids in the repeat region. Upon sequencing of genomic clones, the extra repeat was shown to be encoded by a separate exon, now known as exon 10. This work uncovered the existence of at least two types of tau isoforms in the human brain, with three repeats (3R tau) or four repeats (4R tau) of a conserved tubulin-binding motif [44]. Sequencing of a large number of cDNA clones revealed the existence of additional tau isoforms with two (29-N1, and 59 amino acid-N2) inserts in the N-terminus region (due to alternative splicing of exons 2 and 3), in combination with both three and four repeats (Figure 1). With the isoforms described previously, this gave a total of six human brain tau isoforms ranging from 352 to 441 amino acids in length [45]. The primary sequence of the longest tau isoform is shown in Figure 2. The most prominent expression of tau was observed during fetal development, when only the shortest (referred to as fetal tau) isoform (N0R3) is expressed (352 amino acids with molecular weight of 45 kDa), while the adult human brain expresses all isoforms with R4 to R3 ratio equal to 1 [46]. Relative amounts of N0, N1 and N2 tau isoforms are 37%, 54% and 9%, respectively [46]. There is a further larger transcript of tau that encodes for a protein of 110 kDa with an additional 254 amino acids in the N-terminal projection arm, but this protein is generally restricted to the peripheral nervous system [47].

**Figure 3:**
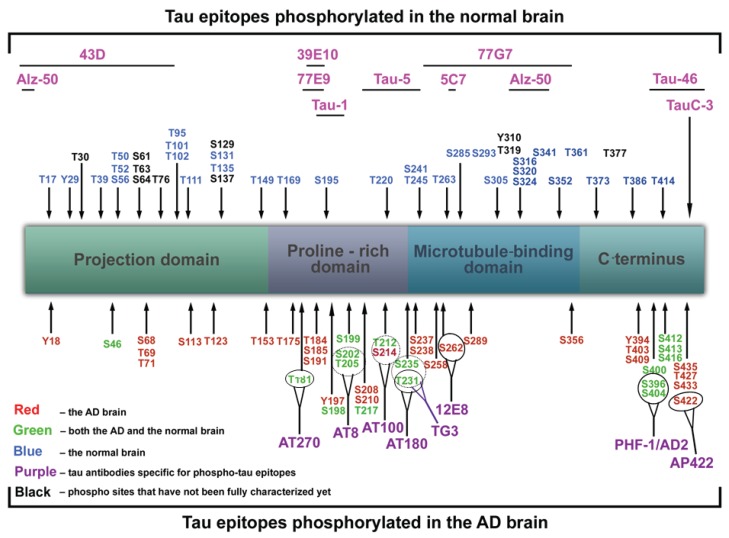
Putative phosphorylation sites on tau protein and epitopes specific for major tau antibodies. Red color denotes amino acids phosphorylated in AD brain, green in both AD and normal brain, blue in normal brain, while black color means that those phosphorylation sites have not been fully characterized yet. Tau antibodies specific for phospho-tau epitopes are given in purple, while pink color denotes antibodies specific for non-phosphorylated tau epitopes: Alz-50 (aa 2–10, aa 312−342), 43D (aa 1–100), 77E9 (aa 185–195), 39E10 (aa 189–195), Tau-5 (aa 210–230), 5C7 (aa 267–278), Tau-1 (aa 195, 198, 199 and 202), 77G7 (aa 270–375), Tau-46 (aa 404–441), TauC-3 (tau cleaved on aa 421). Red—in the AD brain; Green—in both the AD and the normal brain; Blue—in the normal brain; Black—phosphorylation sites that have not been fully characterized yet; Purple—tau antibodies specific for phospho-tau epitopes; Pink—tau antibodies specific for unphosphorylated tau epitopes.

Enriched preparations of PHF from extracts of homogenates from AD brains by using N-lauroylsarcosine (sarkosyl) and 2-mercaptoethanol, after removal of aggregates by microfiltration, sucrose density centrifugation and immunoblotting, revealed three tau bands of 60, 64, and 69 kDa ([49]; a minor fourth band of 72 kDa being described later by Mulot *et al.* [50]). This finding fitted well with the previous findings obtained using monoclonal [51] and polyclonal tau antibodies [52]. Epitopes recognised by tau antibodies and phosphorylation sites on tau protein are shown in Figure 3. In 1991, Lee and collaborators purified PHF using a method comparable to that of Greenberg and Davis [53], and using protein chemical analysis claimed that they are made entirely of full-length hyperphosphorylated tau protein [53].

In 1992, it was shown that, after dephosphorylation, the PHF-tau bands aligned with the recombinant tau isoform mixture, indicating that PHF-tau consists of all six tau isoforms in a hyperphosphorylated state [46]. These studies led to the widely quoted view that PHF are composed entirely of full-length hyperphosphorylated tau protein. This view was challenged by the Wischik group who, using another monoclonal antibody (mAb 7.51), also raised against enriched proteolytically stable core PHF and recognizing all tau isoforms, showed that phosphorylated tau protein released from PHF preparations by the sarkosyl method was not quantitatively related to the total PHF-tau protein content present in these preparations, whether these were prepared with or without exogenous proteases [54,55]. Indeed the proportion of full-length, phosphorylated tau protein was found biochemically to account for less that 5% of total PHF-tau in the bulk PHF fraction prepared without protease, and less than 15% in the sarkosyl PHF preparation. Although PHF isolated without protease digestion can be immunolabeled by tau antibodies directed against phosphorylation-dependent epitopes located in the N-terminal half of the molecule, this immunoreactivity is lost after proteolytic removal of the fuzzy coat [30,31]. The fuzzy coat consists of the lengthy N-terminal portions of tau molecules that cover the surface of the filaments and are readily sensitive to proteolytic digestion. Such digestion leaves intact the proteolytically stable core structure comprising the left-handed helical or straight ribbon of repeated C-shaped subunits. In other words, the fuzzy coat comprising phosphorylated tau does not contribute to the structural core of the PHF. Core PHF have a mass of 65 kD/nm, whereas 90% of PHF isolated without proteases is 77 kD/nm, with a further 10% having a mass of 110 kD/nm [31]. The core PHF therefore accounts for ~85% of the mass of the filament, with a variable addition of fuzzy coat material which contributes ~12 kD/nm.

The tau species identified in the 12-kD species isolated from the PHF core were found to be derived from a mixture of fragments originating from both 3- and 4-repeat isoforms, but restricted to the equivalent of three repeats in length. There are two distinct species originating from the 4R isoform of tau: the fragment derived from repeats 1, 2 and 3 and the fragment derived from repeats 2, 3 and 4. There is also a fragment derived from the 3R isoform (which lacks the second repeat), comprising the equivalent of repeats 1, 3 and 4. Since all of these fragments are restricted to the equivalent of three repeats in length, they all have identical gel mobility [56]. Therefore, the PHF is composed of a structural core of repeating transverse C-shaped subunits. The only tau protein found within the subunit structure is restricted to the repeat domain of the tau molecule. The contribution of phosphorylated tau is variable and restricted to the fuzzy outer coat of the PHF, accounting for approximately one in seven of the tau molecules found in the PHF [57].

### 1.5. Functions of Tau Protein

Tau protein is most abundantly expressed in axons of central nervous system neurons [13] but can also be found in the somatodendritic compartment of neurons, oligodendrocytes, and non-neural tissues [58]. Probably the most important role of tau protein is to promote assembly and stability of MT [7,8], although this function is complemented by other MAP (especially by MAP1B), as tau knockout mice are viable, fertile, and relatively normal, with no signs of neurodegeneration. Also, knockdown of tau with small interfering RNA does not kill primary neurons in culture or prevent axon formation [59]. Additionally, MAP1B is probably more important for MT stability than tau itself because knockout of *MAP1B* results in abnormal brain development and early death, and concurrent knockout of both *MAP1B* and *MAPT* worsens the phenotype [60].

The most common post-translational modifications of tau proteins are phosphorylation and *O*-glycosylation [61]. Phosphorylation changes the shape of tau molecule and regulates its biological activity. Most of the phosphorylation sites are on Ser-Pro and Thr-Pro motives, but a number of sites on other residues have also been reported [62,63]. The majority of tau-based therapeutic strategies against neurodegeneration have focused on modulating tau phosphorylation, given that tau species present within NFT are hyperphosphorylated. *O*-glycosylation is characterized by the addition of an *O*-linked *N*-acetylglucosamine (*O*-GlcNAc) on Ser or Thr residues in the vicinity of Pro residues. It is presumed that glycosylation may have a role in subcellular localization and degradation of tau proteins [64]. The recent discovery that tau is also modified by acetylation requires additional research to provide greater insight into the physiological and pathological consequences of tau acetylation [65].

Tau protein can be divided into two main functional domains: the basic MT binding domain (towards the C-terminus) and the acidic projection domain (towards the N-terminus) [66]. The MT binding domain regulates the rate of MT polymerization through highly conserved repetitive domains R1–R4 encoded by exons 9–12 [36]. Adult tau isoforms with 4R (R1–R4) are about 40-fold more efficient at promoting MT assembly than the fetal isoform that is lacking exon 10 and thus having only 3R (Figure 1) [67]. The absence of expression of the R1–R2 inter-repeat region during fetal development allows for the cytoskeletal plasticity required of growing immature neurons and their elongating axons [64]. Apart from binding to MT, the repeat domains of tau also bind to tubulin deacetylase, histone deacetylase 6 (HDAC6) [68] and apolipoprotein E (apoE, more with the ε3 than the ε4 isovariant [69]).

The projection domain is so called because ultrastructurally it appears as a filamentous “arm” projecting from the wall of the MT. In recent years, many hitherto unknown binding partners of the projection domain have been identified. The projection domain of tau may be involved in cell signaling that occurs through the interaction with Lck, Fgr and cSrc (Src-family kinases), growth factor receptor-bound protein 2 (Grb2), phospholipase C-γ [70], phosphatidylinositol and phosphatidylinositol bisphosphate [71,72], peptidyl-prolyl cis/trans isomerase Pin 1, and many others (for review see [73]), making them potential therapeutic targets in tauopathies [74]. In synapses, the projection domain of tau interacts with protein kinase Fyn (plays an important role during myelination [75]), postsynaptic density protein 95 (PSD-95) [76], and *N*-methyl-d-aspartate receptors (NMDAR). Tau knockout mice show that tau is essential for NMDA-dependent long-term potentiation (LTP) and α-amino-3-hydroxy-5-methyl-4-isoxazolepropionic acid (AMPA)-dependent long-term depression [77,78,79]. The function of tau protein in the response to heat stress in the cell is also worth noting. When the heat stress occurs, tau protein binds to DNA and enhances DNA repair [80]. An additional “knot” of tau being entangled in epigenetic landscape of neurodegeneration comes from the finding that by acting as a HDAC6 inhibitor, tau is being indirectly involved in both (dys)regulation of transcriptional activity and impairment of autophagic clearance by the ubiquitin proteasome system [81,82].

### 1.6. Amyloid Cascade Theory

The so-called “cholinergic hypothesis of AD” [83,84] dominated the late 1970s and early 1980s, and the “calcium hypothesis” in the late 1980s. After the milestone discovery that cerebrovascular amyloid and NP are composed of Aβ (as they shared the same antigenic determinants; [85,86]) in both AD and Down syndrome [87], and that the V717I missense “London” mutation in the amyloid precursor protein gene (*APP*) on chromosome 21 was found to be causally related to the early-onset autosomal-dominant familial AD [88], Hardy and colleagues [89,90] proposed the “amyloid cascade hypothesis”, which has become a dominant driver of AD research ever since. According to the amyloid theory, excessive production of Aβ via serial cleavage of the larger amyloid precursor protein (APP) molecule by β-secretase (β-site APP cleaving enzyme, BACE, encoded by the *BACE1* gene) and γ-secretase (multiprotein complex now known to consist minimally of four individual proteins: presenilin, nicastrin, anterior pharynx-defective 1, APH-1, and presenilin enhancer 2, PEN-2; [91,92,93]), is the key pathological event which drives all other pathological changes (astrocytosis, microglial activation, neuronal death, synaptic loss, and the development of NFTs and dementia) not only in early-onset familial cases but also in late-onset, sporadic cases of AD. In 1987 Goldgaber and collaborators isolated APP and localized its gene to chromosome 21 [94]. It should be noted here that the first *APP* mutation discovered was actually the G to C mutation at codon 693 (APP E693Q) that was not associated with AD, but rather with hereditary cerebral hemorrhage with amyloidosis—Dutch type (HCHWA-D; [95,96]). Interestingly enough, out of four other known mutations within the Aβ part of *APP* (exons 16 and 17) two also cause fatal hemorrhage due to amyloid angiopathy (APP C692G-Flemish and APP E693K-Italian), while only rare “Arctic” (APP E693G) and Osaka (APP E693Δ) mutations cause early-onset AD (EOAD). The well-known fact that many families exist in which AD has an early onset (before age of 60) and is inherited in an autosomal dominant manner [97] could not be explained by a very small number of AD families in which *APP* mutations were found. This question was resolved in part by the discovery of mutations in the presenilin 1 gene (*PSEN1*) on chromosome 14 [98,99], and its homologous presenilin 2 gene (*PSEN2*) on chromosome 1 [100,101]. These mutations of *PSEN* genes further strengthened the amyloid theory, but the pathogenesis of AD remained elusive. Further research showed that PSEN1 and PSEN2 are part of the γ-secretase complex, which cleaves APP at several points resulting in Aβ of various lengths: the lengths associated with AD are 40 and 42 amino acids long with Aβ_42_ more likely to aggregate to form SP in the brain than Aβ_40_. All *PSEN* mutations lead to an increase in the Aβ_42_:Aβ_40_ ratio, although the total quantity of Aβ produced remains constant [102,103]. This can come about by various effects of the mutations of γ-secretase. Presenilins are also implicated in the processing of notch [104,105], an important developmental protein (mice that have *PS1* knocked out die early in development from developmental abnormalities similar to those found when notch is disrupted, [106]). APP can also be cleaved by α-secretases such as a disintegrin and metalloproteases domain 10 (ADAM10) and tumor necrosis factor alpha (TNF-α) converting enzyme (TACE), although this cleavage does not result in Aβ but instead generates APPs-α, which are thought to be neuroprotective [107].

Collectively, the genetic etiology of AD is very complex: early-onset AD (less than 5% of cases) is often familial (fAD) with autosomal dominant and fully penetrant inheritance and can be caused by any of more than 200 pathogenic mutations in *APP* (33 mutations, duplication), *PSEN1* (185 mutations) and *PSEN2* (13 mutations; http://www.molgen.ua.ac.be/ADmutations). Most AD cases (over 95%) however are sporadic, late-onset (sAD, LOAD) and have less evident genetic components. The ε4 variant of the gene encoding apolipoprotein E (*APOE*) is known to confer increased risk for LOAD [108,109] with partial penetrance. Based on 320 meta-analyses of 1395 studies in which 695 genes and their 2973 polymorphisms have been tested as late-onset AD candidate genes, over 30 yielded positive evidence for association. The number one gene is *APOE*, with a Bayes factor (BF) > 50. Using *APOE* genotype ε3/ε3 as a neutral benchmark for comparison, individuals with a single copy of the ε4 allele manifest a 5 fold increased chance of developing LOAD, while those with two copies have an estimated 20 fold increased risk [110]. It seems that different *APOE* alleles are not associated with an increase in Aβ production, but with a reduced ability to clear Aβ from the brain [111,112]. This may be related to decreased production of Aβ auto-antibodies in AD subjects [113]. The next nine genes with the highest association with LOAD are: *BIN1* (BF = 23.4) that encodes several isoforms of a nucleoplasmic adaptor protein, one of which was identified as MYC-interacting protein, *CLU* (BF = 20.1) that encodes apolipoprotein J, *ABCA7* (BF = 18.8) for ATP-binding cassette, subfamily A [ABC1], member 7, *CR1* (BF = 18.1) for complement component receptor 1, *PICALM* (BF = 17.3) for phosphatidylinositol-binding clathrin assembly protein, *MS4A6A* (BF = 8.7), *CD33* (BF = 7.7) for a transmembrane receptor expressed on cells of myeloid lineage-cluster of differentiation 33, *MS4A4E* (BF = 6.9) coding for protein membrane-spanning 4-domains, subfamily A, member 4E, and *CD2AP* (BF = 6.6) that codes for a scaffolding molecule that regulates the actin cytoskeleton (according to www.alzgene.org accessed on 11 February 2015). Genetic variants of all of these genes have a relatively minor influence on AD progression when altered [114] and their influence on the development and course of sAD remains largely unknown [115,116,117]. Most recently, rare mutations of *TREM2* [118] and *PLD3* [119] have also been discovered to confer a much larger increase in risk for LOAD than the aforementioned common sequence variants [120].

### 1.7. Staging of Tau Pathology

During the 1990s, the significance of tau pathology for neurodegenerative diseases, in particular for AD, remained in the shadow of the amyloid theory. However, as the distribution pattern and overall quantity of Aβ turned out to be of limited significance for pathological staging of AD progression and symptom severity, and after detailed studies of the maturation and distribution of NFTs showing correlation with degree of cognitive decline and memory impairment in AD, Braak and Braak proposed a neuropathological staging of the gradual deposition of abnormal tau within vulnerable neurons in brain areas in the form of either NFT or neuropil threads (NT). At first they used classical silver staining [66] and later immunohistochemical staining for hyperphosphorylated tau using antibody AT8 [67]. The finding that NFT provide a better association with cognitive impairment was confirmed by other researchers [121,122], supporting a significant role for tau pathology in the disease. The Braak staging system classified the topographic progression of AD neurofibrillary degeneration into six stages, spreading from the transentorhinal region to the hippocampal formation (initial stages I and II, which clinically correlate with subjective or objective impairment of memory for recent events and mild spatial disorientation, but with preservation of general cognitive functioning with or without minimum impairment of activities of daily living), then to the temporal, frontal, and parietal neocortex (intermediate stages III and IV, which correlate with impaired recall, delayed word recall and word finding difficulties, disorientation in time and space, and impaired concentration, comprehension and conceptualization among other symptoms of dementia), and finally to unimodal and primary sensory and motor areas of the neocortex (late stages V and VI, which roughly correlate with disturbances in object recognition, and other perceptual and motor skills).

Besides the fact that Braak and collaborators showed that AD-related pathology proceeds in strictly defined stages, based on the notion that NFT evolve from an accumulation of abnormal tau without PHF formation (described as the “pre-tangle” stage, [123]) they also proposed that abnormal phosphorylation is a crucial step leading to the formation of both soluble and insoluble tau filaments [124], that neuronal damage in AD actually starts many years before any clinical symptoms and signs and that, unlike Aβ, the distribution of tau pathology is associated with the clinical progression of AD. In contrast to the amyloid cascade hypothesis of AD, which implies that tau pathology is a secondary, downstream phenomenon, the neuropathological findings of Braak and collaborators have fueled a significant controversy concerning the importance or contributions of Aβ burden in producing damage compared to that caused by tau pathology. Additionally, in AD, the pathological Aβ and tau proteins mutually interact and are influenced by many other contributors, such as inflammatory [125], vascular, and environmental factors, as well as compensatory neuroplastic responses to counteract neural injury associated with neurodegenerative processes [126], all of which may promote cognitive and behavioral decline.

### 1.8. Mutations in MAPT Gene and Tauopathies

In the late 1980s and early 1990s, evidence implicating tau pathology in neurodegenerative diseases other than AD began to emerge. As early as 1986, Pollock and colleagues reported that the filamentous aggregates in Pick’s disease (now part of the group of disorders classes as frontotemporal lobar degeneration, FTLD, and called frontotemporal dementia, FTD), progressive supranuclear palsy (PSP) and AD shared the antigenic determinants of tau [127]. While hyperphosphorylation of tau is a feature common to all of these diseases, unlike AD, they lack significant Aβ and α-synuclein pathology. However, biochemical differences in the tau isoforms isolated from preparations of the pathological filaments in various tauopathies were observed. All six tau isoforms are present in sarkosyl extracts in equal ratios of R3 and R4 isoforms is observed in class I tauopathies, which are biochemically characterized by tau triplets of 60, 64 and 69 kDa, and additional minor bands of 72/74 kDa. Such a profile is characteristic for AD, some cases of frontotemporal dementia and parkinsonism linked to chromosome 17 (FTDP-17), Niemann-Pick disease type C, Down syndrome and dementia pugilistica [128]. On the other side, sarkosyl extracts from the filaments of PSP [129], corticobasal degeneration (CBD; [130]), argyrophilic grain disease (AgD; [131]), and some cases of FTDP-17, contain tau protein that separates as doublets of 64 and 69 kDa and are predominantly composed of tau isoforms with 4R (class II tauopathies), whereas sarkosyl extracts from filaments of Pick’s disease are characterized by the presence of pathological tau doublets of 60 and 64 kDa and contain mainly 3R tau isoforms (class III tauopathy). Class IV tauopathy is represented by a single neurological disorder—myotonic dystrophy type I (DM1) or Steinert’s disease, in which a major insoluble tau band of 60 kDa, and minor 64 and 69 kDa bands have been identified [48,61,76,128,132,133]. Despite the fact that these studies showed the filaments contain tau, they did not provide much direct information about the relevance of tau dysfunction and filament formation in the neurodegenerative disease process. Although tau involvement in neurodegenerative diseases other than AD attracted wide attention, genetic evidence linking dysfunction of tau protein to neurodegeneration and dementia had been missing. In 1994, Wilhelmsen and colleagues reported linkage of an autosomal dominantly inherited form of FTD with parkinsonism and amyotrophy (disinhibition-dementia-parkinsonism-amyotrophy complex, DDPAC) to chromosome 17q21.2, the region that contains the *MAPT* gene [134]. In 1997, Spillantini and colleagues first used the term tauopathy to describe “multiple system tauopathy with presenile dementia” (MSTD), where tau filaments contain 4R isoforms in absence of 3R tau [135]. In parallel, Murrell and colleagues showed that the genetic defect in MSTD mapped to chromosome 17q21-22 [136]. At that time, 13 kindreds were considered to have sufficient evidence of linkage to be included in what was then named “frontotemporal dementia and parkinsonism linked to chromosome 17” (FTDP-17; [137]). The exclusive presence of 4R tau in the MSTD filaments naturally led to an examination of the isoform composition of the pool of soluble tau and its findings suggested that increased splicing of exon 10 of the *MAPT* gene might be the cause of familial MSTD. Upon DNA sequencing, a guanine (G) to adenine (A) transition at position +3 of the intron following exon 10 was found, which segregated with disease [138]. At that time, an additional eight mutations in the *MAPT* gene had been reported by two other groups: Poorkaj *et al.* reported two exonic mutations (P301L and V337M) in two families with FTDP-17 [139], while Hutton *et al.* reported six different mutations in 10 families: three of these mutations (G272V, P301L and R406W) were missense mutations in exons, while the other three were in the 5' splice site of exon 10 [140]. Later that year, missense mutations were shown to reduce the ability of tau to promote microtubule assembly [141,142].

The discovery of these and other subsequent mutations in the *MAPT* gene finally confirmed that molecular tau pathology can give rise to neurodegeneration in the absence of Aβ changes and that tau is in a central position as a key pathological component (leading from normal, soluble tau to abnormal, filamentous tau which causes neurodegeneration and dementia) across many neurodegenerative states and disorders, either through mutations in the *MAPT* gene or the effects of upstream stressors such as Aβ or oxidative damage (for review, see [143]). Why particular neurons are susceptible to the buildup of misfolded tau and tau aggregation remained unanswered, leading to much research activity on tau, with the development of transgenic animals and cell lines to model the effects of expressing disease-related mutations of the *MAPT* gene. The level of biochemical diversity and, consequently, complexity of tau pathology is perhaps best illustrated by the simple fact that specific *MAPT* mutations are associated with specific forms of FTD; in contrast, the very same mutation (such as TAU P301L) can apparently lead to either CBD or FTDP-17 in the same family, suggesting that other factors (genetic, epigenetic, environmental) may influence which neurons are affected and when this occurs. Several other findings have further emphasized the importance of tau in neurodegeneration (reviewed in [144]). A *PSEN1* mutation causes a Pick’s disease phenotype including FTD tau pathology without deposition of Aβ [145]; some *MAPT* single nucleotide polymorphisms have also been linked to sporadic Parkinson’s disease (PD, [146]); and retarded axonal extension in tau-deficient hippocampal neurons may be due to reduced MT transport by lack of tau-mediated regulation of motor protein activities [147].

## 2. Tau Protein Pathological Changes in Primary and Secondary Tauopathies

### 2.1. Mechanisms

**Figure 4:**
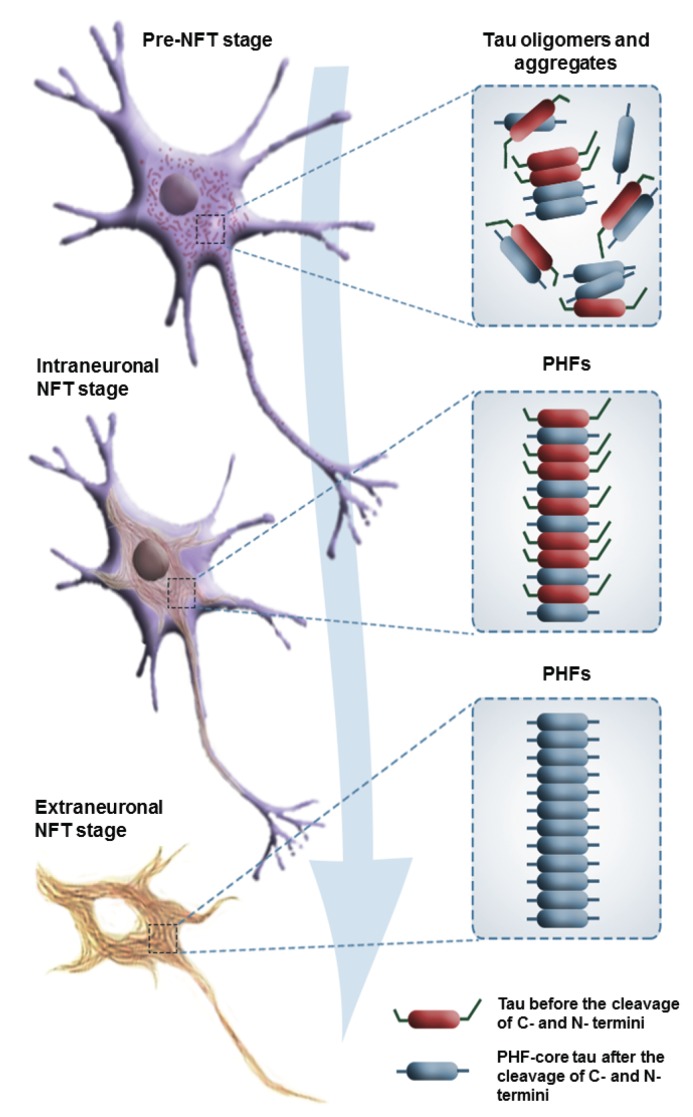
The sequence of cytoskeletal changes due to the pathology of tau protein divided into three stages: pre-tangle (pre-NFT) stage, and intraneuronal and extraneuronal stages. See text and Figure 5 for details.

As mentioned earlier, compelling evidence that tau malfunction or dysregulation alone can be sufficient to cause neurodegeneration came in 1998 from the identification of mutations in the *MAPT* gene on chromosome 17 that causes frontotemporal dementia with parkinsonism (FTDP-17) [140], making cytoskeletal abnormalities a pivotal mechanism in neurodegeneration in AD (mutations in the *MAPT* gene cause primary tauopathies, while AD is the most important secondary tauopathy with the *MAPT* gene itself not being mutated) [143,148]. More specifically, abnormal phosphorylation, aggregation, and proteolysis of the tau protein in a “pre-tangle” stage of neurofibrillary degeneration (Figure 4) has been neuropathologically documented to be an early and crucial event in the pathogenesis of AD, but also other sporadic tauopathies, such as AgD [131] and PSP. Historically, NFT were considered indicators of cell death, particularly given that since 1995 they have been consistently shown to correlate well with the severity of dementia in AD, in contrast to Aβ plaque deposition does not [122]. However, which variety of tau is the most toxic (aggregated misfolded/fibrillar, soluble hyperphosphorylated/mislocalized, or both) and whether that toxicity represents a gain or loss of function remains an unanswered question. As there is little direct evidence that tau fibrils themselves are toxic, the hypothesis that soluble oligomeric forms of tau are more toxic to neuronal and synaptic function is increasingly gaining favor. The formation of NFT may actually protect neurons acutely from the effects of toxic soluble tau, as shown by Kopeikina and collaborators [149].

The tau fragment first isolated from the PHF core is approximately 100 amino acids in length. Its N-terminus was defined by sequence analysis [30,56], and its C-terminus was defined by epitope mapping of MN423. Immunoreactivity was shown to depend on a specific C-terminal trunctation at Glu391 [33,150]. Thus, the N-termini of the tau fragments found in the proteolytically stable structural core of the PHF are located 15-residues C-terminal to the start of the repeats, and have a characteristic C-terminal truncation at position Glu391 which is 15-residues C-terminally to the end of the repeats [151]. These features explained the paradox noted earlier, namely that tau protein isolated from the core of the PHF is not necessarily recognized by anti-tau antibodies (if these are directed against epitopes located in the N- or C-terminal portions of the molecule, whether or not phosphorylated), and the monoclonal antibody raised against the PHF core does not recognized normal full-length tau protein [30].

**Figure 5:**
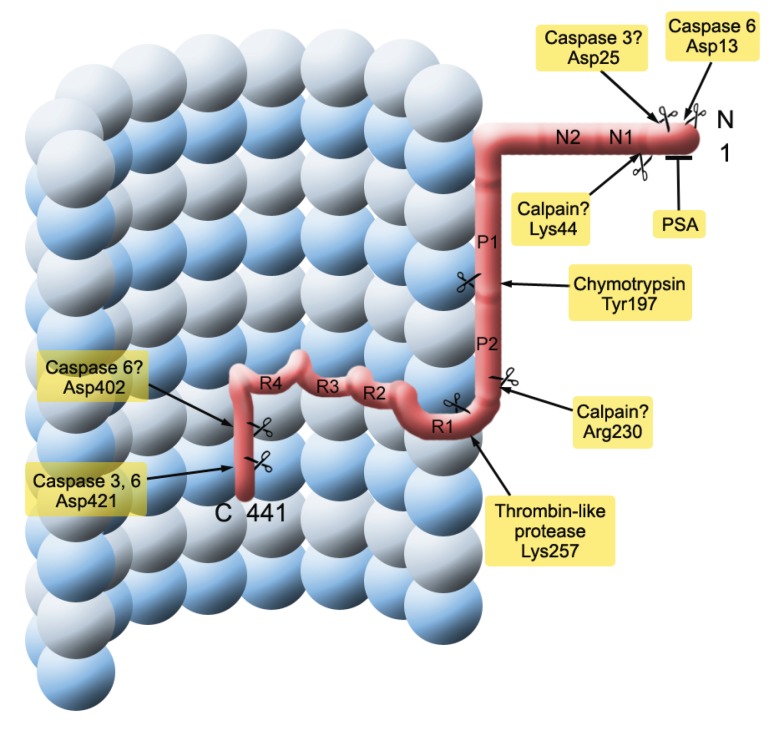
Diagram showing sites for potential cleavage of tau protein. The sequential cleavage of the tau protein leads to the formation of the tau protein fragment from the microtubule-binding repeat region (see text and Figure 4). Tau cleavage is more likely to take place while protein is unbound to microtubules, either aggregated to itself or associated with proteins other than tubulin. PSA = puromycin-sensitive aminopeptidase.

The characteristic N- and C-terminal truncations found in the core PHF-tau unit are exactly 3-repeats apart, but shifted by 15-residues with respect to the N- and C-terminal extent of the normal repeat domain. This feature was shown to represent the footprint of a pathological tau-tau binding interaction, which can occur through the repeat domain of the tau molecule and which locks it into a characteristic proteolytically stable configuration. The Glu391 trunctation could be reproduced *in vitro* after binding of full-length tau to a fragment terminating at Ala390 and lacking immunoreactivity to MN423 [152]. Surprisingly, when the bound complex was taken through repeated cycles of digestion with protease and re-incubation of full-length tau, N-terminal tau immunoreactivity was eliminated in every cycle, whilst a progressive build-up of Glu391 immunoreactivity detected by MN423 was observed. Thus, the repeat domain of tau is able to catalyze and propagate the conversion of normal soluble tau into the aggregated and truncated oligomeric form in a cell-free setting.

The same phenomenon has recently been demonstrated within the physiological milieu of the cell. Although the repeat domain fragment is highly toxic when expressed in cells, cells can be maintained provided expression levels remain very low, where it acts as a latent primer or seed. When such cells were co-transfected with inducible, full-length 4R tau, it was possible to induce tau aggregation and template-directed truncation of full-length tau in a controlled manner [153]. The neo-fragment generated in a concentration-dependent manner with respect to induced full-length tau proved to be the core tau unit itself. Thus the repeat domain has the ability to define a template-directed truncation of full-length tau to reproduce and amplify the proteolytically stable species characteristic of the PHF core in AD recruiting normal tau in the process. Importantly, the process does not require abnormal phosphorylation, or any other post-translational modification once it has been initiated. The same phenomenon has been reported independently in transgenic rat studies expressing a truncated tau species containing the repeat domain [154,155]. Insoluble tau aggregates were found to form in the brain consisting of both transgenic human truncated tau and endogenous rat tau in a 1:1 ratio. A further suprising feature in the cell-model was the demonstration that when full-length tau is induced in the presence of the truncated tau primer, it is unable to bind normally to microtubules, but instead is preferentially directed to the aggregation/truncation pathway. In contrast, full-length tau expressed in the absence of the truncated tau primer showed normal tubulin binding [153]. These results are consistent with that the pathological tau-tau binding affinity through the repeat domain is higher than the normal tau-tubulin binding affinity [57]. Thus, it is unnecessary to invoke pathological phosphorylation as a primary mechanism to account for loss of normal microtubule binding in AD. Rather, the almost complete redistribution of the tau protein pool from soluble/tubulin-bound to insoluble/aggregated that occurs in AD is simply a kinetic consequence of the properties of the pathological tau-tau binding interaction of the repeat domain.

The truncated core tau unit of the PHF has also been termed “the F3 fragment” (Figure 5) [156]. The F3 fragment in the oligomeric state is resistant to cytosolic proteases and may therefore be transported unchanged to axon terminals where it may not only damage synapses, but from where it may propagate between neurons either trans-synaptically or by exosomes, thus initiating the same neurofibrillary cascade in previously healthy neurons [157]. As recently reviewed by Jadhav and collaborators [76], several factors point strongly towards a prominent role of presynaptic tau protein in mediating synaptic pathology, including (1) cognitive decline that best correlates with synaptic loss and synaptic failure; (2) synapse loss in parallel with NFT formation and occurring in the same regions in AD brains; and (3) higher NFT count associated with lower levels of presynaptic proteins in AD. In a prospective study three different synaptic protein (synaptophysin, SNAP-25 and syntaxin) were found to be progressively in neocortex at Braak stages III-VI [158], NFT-bearing neurons demonstrating, for example, a 35%–57% reduction in synaptophysin mRNA in AD brain [159]. Last but not least, synaptic deficits are observed in FTLD [160], PSP, and Niemann-Pick disease type C [161], which are all independent of any Aβ pathology. Moreover, tau proteomic changes are also confirmed in several tau transgenic models (for review, see [76]).

Tau fragments are also able to propagate between neurons trans-synaptically, causing the spread of neurofibrillary degeneration to post-synaptic neurons [162]. In this case, mutations in the *APP*, *PSEN1* and *PSEN2* genes in familial AD only initially compromise endosomal-lysosomal processing and mitochondrial metabolism by altering Aβ clearance thus activating caspases responsible for tau cleavage or providing seeding factors required to nucleate pathological aggregation of tau protein through the repeat domain.

### 2.2. Seeding and Spreading of Tau Proteins

**Figure 6:**
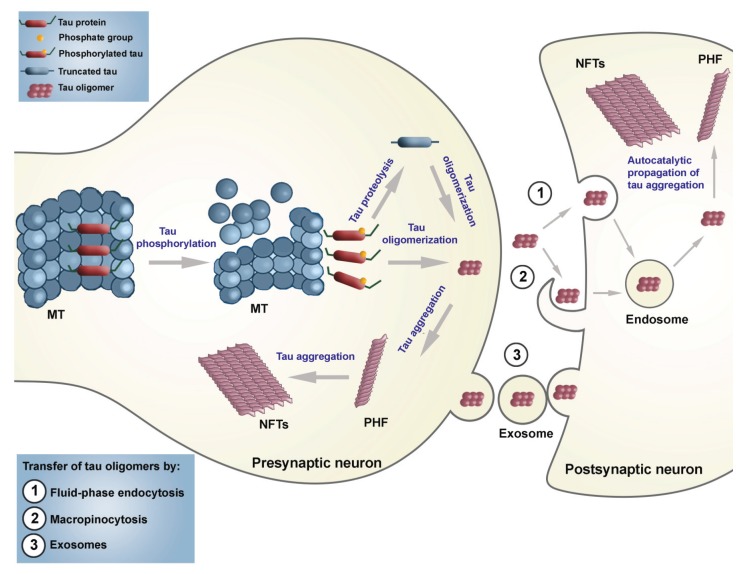
Schematic representation of three different ways of anterograde spreading of tau aggregates by endocytosis, macropinocytosis, and exosomes.

Tau can be directly involved in the spread of AD pathology to neighbouring neurons. However, direct evidence for molecular mechanisms supporting this hierarchical progression (“prion-like behaviour of misprocessed tau”) has remained elusive [163]. The most recent data obtained indicate that tau pathology indeed may be induced and propagated after the injection of tau oligomers or aggregates in either wild-type or mutated *MAPT* transgenic mice [164], and that tau aggregates can be transferred from cell to cell *in vitro* [164,165] and *in vivo* [166,167]. These new findings suggest that suppressing the spread of tau oligomers could be a target for development of disease-modifying therapeutics for AD and other tauopathies, although further studies are needed to determine whether pathologic tau oligomers spread trans-synaptically or by exosomes. Additionally, it was proved that antibodies blocking tau aggregate seeding improve cognition *in vivo* [168]. In the case of soluble monomeric or small oligomeric tau protein, the endocytosis appears to be clathrin-dependent (reviewed in [169]). In contrast, larger aggregates of tau could bind heparin in the extracellular matrix and be internalized through macropinocytosis [170]. As a result of exocytosis and endocytosis, the spreading of tau can occur in various neurodegenerative diseases (tauopathies) including AD. Three plausible mechanisms of tau spreading are shown schematically in Figure 6. Additionally, it appears that microglial cells may facilitate tau propagation by phagocytosis and exocytosis of tau protein [171]. Avila *et al*. (2015) have speculated on how the top six LOAD risk genes (*APOE*, *BIN1*, *CLU*, *ABCA7*, *CR1* and *PICALM*), that all interact with tau, may be involved in the transmission of tau [172].

In one of our previous pilot studies, we observed evidence suggesting the spreading of tau pathology in the brains of patients who suffered from mild cognitive impairment (MCI) with antibodies specific for phospho-tau epitopes Ser202 and Thr205 (AT8), Ser396 (AD2) and PHF core (MN423). In early MCI, only AT8 immunoreactivity was observed in the neurons of the superficial entorhinal cortex layers and temporal isocortex. In more advanced MCI cases, we detected both AT8 and AD2 immunoreactivity in these neurons. Immunoreactivity to MN423 in layers II-III of the entorhinal and temporal isocortical neurons was observed only in advanced MCI and early AD patients. Concurrently, AT8 and AD2 immunoreactivity was found in neurons of layers II, III and V of the entorhinal cortex and AT8-immunoreactive pyramidal neurons were already present in layers III and V of the temporal isocortex. As an increase in MN423 immunoreactivity positively correlated with Braak stages, these findings support the concept of Wischik and collaborators [157] that cycles of proteolytic removal of N- and C-termini of tau are followed by stepwise binding of further tau in an autocatalytic process and that the repeat domain of tau is able to catalyse and propagate the conversion of normal soluble tau into accumulations of the aggregated and truncated oligomeric form.

### 2.3. Therapeutic Approaches Targeting Tau Protein Processing in Tauopathies

**Figure 7:**
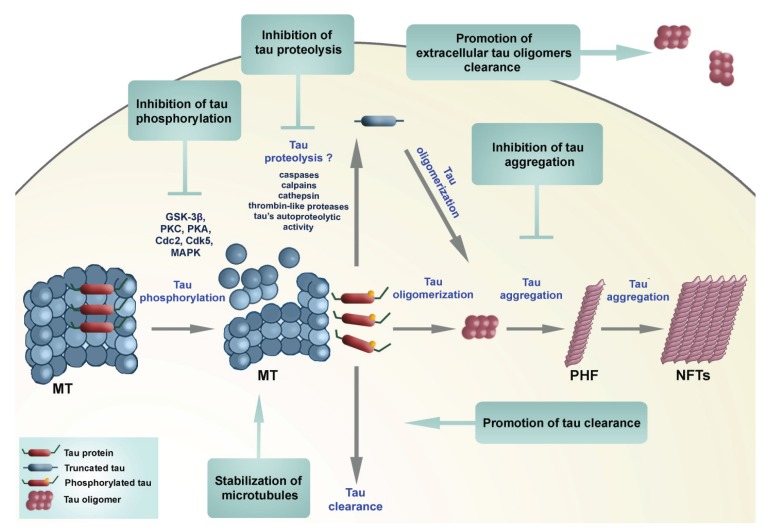
Diagram showing potential neuroprotective strategies to reduce tau aggregates. See text for details.

A number of neuroprotective strategies have been proposed based on the phosphorylation theory of tau pathology (Figure 7).

(1) MT-stabilizing agents, which as an approach does not address the accumulation of toxic tau aggregates.

(2) Modulation of tau phosphorylation has been shown to prevent motor impairments in tau transgenic mice [173]. Green coffee, a non-toxic small molecule, found to be an inhibitor of protein phosphatase 2A methylesterase, was shown to improve cognitive and motor performance in mouse models with tau pathology [174].

(3) A different approach, which does not depend on the phosphorylation theory, is based on selective inhibition of pathological tau aggregation [157]. The problem in identifying suitable tau aggregation inhibitors (TAI) is that most assays for tau aggregation are based on fibril formation (which require relatively high concentrations of tau, tau constructs limited to the MT binding region with the motif necessary for fibril formation, and a facilitator of aggregation, such as heparin). If compounds are selected that dissociate preformed large aggregates into smaller (still toxic) oligomers then they may be detrimental. In these assays, fibril formation is measured by a shift in fluorescence of an intercalating reporter dye binding to β-sheet structures within tau fibrils.

(4) A more promising approach may be to target tau oligomers, whether these are intracellular or extracellular. Purified tau oligomer species have been demonstrated to be neurotoxic (they directly impair synaptic function and long-term potentiation, LTP) *in vitro* in a dose-dependent manner. Extracellular tau levels measured in AD are more than four orders of magnitude lower than intracellular tau concentrations, and as such may represent a more amenable pharmacological target.

(5) A quite different strategy is to target tau clearance—e.g., by rapamycin that induces macroautophagy [175], inhibitors of Hsp90 chaperone protein that binds to misfolded proteins or by immunotherapeutic approaches [176].

(6) Finally, it may be possible to target tau proteolysis directly. Cellular enzymes implicated in tau proteolysis include caspases, calpains, cathepsins and a thrombin-like protease. Regardless of the protease, it is reasonable to presume an irreversible loss of normal function of tau once it is truncated. Disulfide-linked oligomers of tau can be observed in AD brains and cerebrospinal fluid (CSF) samples, and show significant fragmentation, making them great potential targets for early diagnosis of AD [177]. The major advantage of targeting tau proteolysis is that it may be more straightforward to inhibit an enzymatic mechanism than aggregation, provided the proteolytic activity in question can be shown to be rate-critical. It is possible that inhibition of truncation could prevent formation of aggregation-prone fragments and also trans-synaptic/exosomal spread of tau pathology.

**Figure 8:**
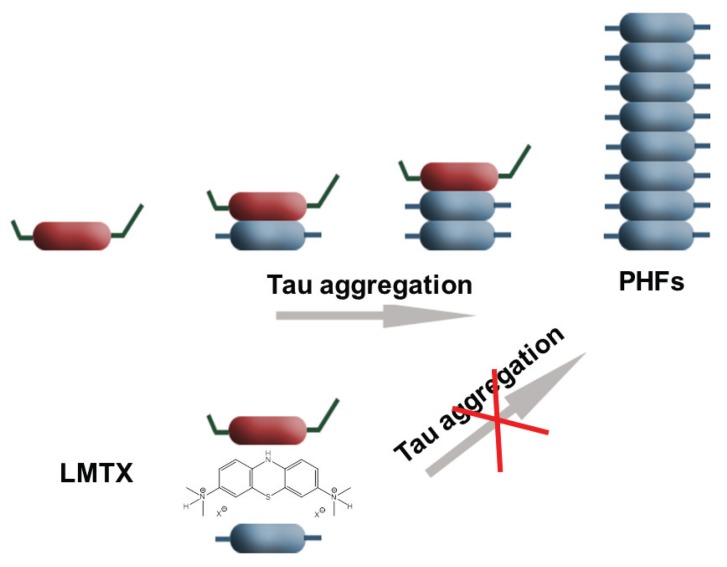
Diagram of tau aggregation inhibitor LMTX (leucomethylthioninium with a suitable counterion), and its presumed mode of action (inhibition of tau aggregation).

Recently, two drugs that targeted tau phosphorylation failed in phase 2 clinical trials [178]. This led Wischik and collaborators [157] to propose that it is not abnormal tau phosphorylation that ought to be reduced by drugs, but tau aggregation. The tau aggregation inhibitor LMTX (leucomethylthioninium with a suitable counter-ion, Figure 8) is currently in three parallel Phase III clinical trials, with the first outcomes expected in 2016 [179,180]. An older form of the molecule (methylthioninium chloride, MTC) was found to have efficacy in mild/moderate AD in a Phase II clinical trial, in which 90% retardation of disease progression could be demonstrated over 12 months [180]. These investigators stressed that hyperphosphorylation of tau may not play a critical role in aggregation of tau and formation of PHF, and that it may even have an inhibitory effect on tau-tau binding. Thus, it might be more important to clarify proteolysis of tau protein (potentially at position Glu391, although this site may simply report the C-terminal extent of the pathological binding domain) that enables the release of the C-terminal fragment. This fragment is the one that appears to be important in the formation or propagation of proteolytically stable tau oligomers that can spread to neighboring neurons trans-synaptically, further propagate tau pathology and lead ultimately to formation of PHF. Recently, it has been proposed that tau protein acetylation may be responsible for tau aggregation in AD. Grinberg and collaborators detected tau acetylation at Lys274 in all tauopathies (both primary and secondary), except in AgD [181]. They hypothesized that tau acetylation could also promote the spreading of tau pathology (whereas in AgD it could have a protective role in this respect).

The investigators are currently putting most of their efforts into basic, preclinical and clinical testing of methylene blue (MB) and its derivatives. MB is a phenothiazine that crosses the blood brain barrier and acts as a redox cycler. Moreover, besides its beneficial properties as being able to improve energy metabolism and to act as an antioxidant, it is also able to reduce tau protein aggregation. How exactly LMTX and MTC exert their neuroprotective effects *in vivo* is not fully understood. MB (as MTC) is able to reduce the amount of sarkosyl-insoluble tau in *Drosophila* that express human wild-type tau [182], to disaggregate PHF isolated from AD brain [152] and to block prion-like processing of tau protein in cell models [153]. Both MTC and LMTX have been shown to reduce tau pathology and reverse behavioural deficits in transgenic mouse models of established pathology based either on the repeat domain fragment or on full-length mutant tau P301S [183]. MB, together with its derivatives (metabolites), azure A and azure B, is able to stimulate protein degradation and inhibit oxidative damage [184] and also inhibit the activity of caspase-1 and caspase-3 [185]. MB given prior to the onset of tau aggregation was also able to prevent learning and memory deficits in tau transgenic mice [186], suggesting a potential preventative utility. Other possible inhibitors of tau aggregation are rhodanine-based inhibitors, phenylthiazolyl-hydrazide inhibitors, *N*-phenylamines, phenothiazines and benzothiazoles, and polyphenols and anthraquinones [187].

## 3. Conclusions

Although the pathogenic nature of the each type of protein deposit has been a controversial issue for many years, it is now increasingly accepted that abnormal forms of tau protein are directly involved in the initiation of neurodegerative processes. This conclusion is based primarily on the discovery that dominant missense mutations in the *MAPT* gene are associated with dominant, familial forms of FTD. Known polymorphisms in *MAPT* which confer susceptibility not only for AD and FTD, but other neurodegenerative diseases as well, together with a possible additional novel disease locus near the *MAPT* gene [188], strongly support the key role of tau protein not only in primary tauopathies but also in the pathogenesis of LOAD and other secondary tauopathies.

Why disease onset takes decades before symptoms occur remains unclear at present, but current results suggest a reduced ability to clear out misfolded, oligomerized and aggregated tau proteins that increase with advancing age. As many drug discovery attempts based on the amyloid cascade hypothesis have proved unsuccessful, and due to advances in our understanding of the role for tau in AD pathogenesis [189], it is safe to conclude that tau protein will become an increasingly important therapeutic target for the future. The results of clinical trials with LMTX are eagerly awaited to confirm whether a treatment for tauopathies is viable.

## Abbreviations

3R tau	tau isoforms with three microtubule-binding repeats
4R tau	tau isoforms with four microtubule-binding repeats
A	adenine
Aβ	amyloid β protein
AD	Alzheimer’s disease
AD2	antibody specific for phospho-tau epitope Ser396
ADAM10	a disintegrin and metalloprotease domain 10
AgD	argyrophilic grain disease
AMPA	α-amino-3-hydroxy-5-methyl-4-isoxazolepropionic acid
APH-1	anterior pharynx-defective 1
APOE	apolipoprotein E
APP	amyloid precursor protein
AT8	antibody specific for phospho-tau epitopes Ser202 and Thr205
BACE	β-site APP cleaving enzyme
BF	Bayes factor
CBD	corticobasal degeneration
CSF	cerebrospinal fluid
DDPAC	disinhibition-dementia-parkinsonism-amyotrophy complex
DM1	myotonic dystrophy type I
EOAD	early-onset AD
fAD	familial AD
FTD	frontotemporal dementia
FTDP-17	frontotemporal dementia and parkinsonism linked to chromosome 17
FTLD	frontotemporal lobar degneration
G	guanine
Grb2	growth factor receptor-bound protein 2
HCHWA-D	hereditary cerebral hemorrhage with amyloidosis-Dutch type
HDAC6	histone deacetylase 6
LMTX	leucomethylthioninium
LOAD	late-onset AD
LTP	long-term potentiation
MAP2	microtubule-associated protein 2
MAPT	microtubule-associated protein tau
MB	Methylene blue
MCI	mild cognitive impairment
MN423	antibody for PHF core
MSTD	multiple system tauopathy with presenile dementia
MT	microtubule
NFT	neurofibrillary tangles
NMDAR	*N*-methyl-d-aspartate receptors
NT	neuropil threads
*O*-GlcNAc	*O*-linked *N*-acetylglucosamine
PD	Parkinson’s disease
PEN-2	presenilin enhancer 2
PHF	paired helical filaments
PSA	puromycin-sensitive aminopeptidase
PSEN	presenilin
PSEN1	presenilin 1
PSEN2	presenilin 2
PSP	progressive supranuclear palsy
sAD	sporadic AD
SDS	sodium dodecyl sulfate
SDS-PAGE	sodium dodecyl sulfate-polyacrylamide gel electrophoresis
SF	straight filaments
SP	senile plaques
TACE	tumor necrosis factor alpha converting enzyme
Tau	tubulin-associated unit
TNF-α	tumor necrosis factor alpha
